# Diet and physical activity advice for colorectal cancer survivors: critical synthesis of public-facing guidance

**DOI:** 10.1007/s00520-024-08797-5

**Published:** 2024-08-22

**Authors:** Anna Fretwell, Christina Dobson, Samuel T. Orange, Bernard M. Corfe

**Affiliations:** 1 Faculty of Medical Sciences, Human Nutrition and Exercise Research Centre, Newcastle University, Newcastle Upon Tyne, NE2 4HH UK; 2 Faculty of Medical Sciences, Population Health Sciences Institute, Newcastle University, Newcastle Upon Tyne, NE2 4HH UK; 3 Newcastle University Centre for Cancer, Newcastle University, Newcastle Upon Tyne, NE2 4AD UK; 4 Faculty of Medical Sciences, School of Biomedical, Nutritional and Sport Sciences, Newcastle University, Newcastle Upon Tyne, NE2 4DR UK

**Keywords:** Colorectal cancer survivor, Diet, Physical activity, Health information

## Abstract

**Purpose:**

Colorectal cancer (CRC) survivors report that diet and physical activity guidance from healthcare professionals following discharge from care is limited. Survivors seek advice from alternative sources. This study critically synthesised the English language diet and physical activity guidance available online for CRC survivors.

**Methods:**

We conducted an internet search to identify national cancer organisations (NCO) in countries with high CRC incidence rates. We searched NCO website content for guidance related to diet and physical activity. Recommendations were categorised by cancer phase (prevention/survivorship), cancer type, and the intended outcome (health or cancer-control–CRC recurrence/CRC-specific mortality). A synthesised guideline was derived from recommendations consistently made by at least half of the sources.

**Results:**

We identified 12 NCOs from six countries, by whom 27 diet and physical activity recommendations were made. For CRC prevention, over 80% of recommendations were aimed at improving cancer-control outcomes. For CRC survivorship, less than 40% of recommendations were aimed at improving cancer-control outcomes. Physical activity was the only recommendation present on more than 50% of NCO websites aimed at improving cancer-control outcomes for CRC survivorship.

**Conclusion:**

Diet and physical activity guidance for CRC survivors on NCO websites is limited and primarily based on recommendations for improving general health, not improving cancer-control outcomes. NCO websites frequently refer survivors to primary prevention guidance, potentially reflecting the lack of evidence specific to CRC survivorship. There is a need for diet and physical activity advice for survivors that is evidence-based, comprehensive, and consistent across organisations and tailored to specific cancer sites.

**Supplementary Information:**

The online version contains supplementary material available at 10.1007/s00520-024-08797-5.

## Introduction

The National Institute of Health and Care Excellence (NICE) recommend that individuals who have survived colorectal cancer (CRC) receive guidance on diet and physical activity prior to discharge from treatment [1]. However, several studies have shown that this guidance is not adequately implemented in clinical practice, and the availability of accessible advice is inconsistent [2–6]. This may be, in part, due to a lack of high-quality evidence to develop diet and physical activity guidelines to support a reduction in the risk of recurrence or mortality amongst CRC survivors [7–11]. The World Cancer Research Fund and American Institute of Cancer Research (WCRF/AICR) published a comprehensive review of diet and physical activity evidence for cancer prevention and survivorship. However, the only recommendation specific to CRC survivors is to follow the recommendations for prevention, if appropriate for their circumstances [7].

Guidelines tailored to specific cancer types and stage of survivorship are critical, as the information and support needs of CRC survivors differ from those of the general cancer population [12–15]. The diagnosis and treatment of all cancers can have a significant impact on physical and mental health, and common sequalae like pain, anxiety, depression, fatigue, sleep problems, and fear of cancer recurrence may continue to effect food choices and physical activity following recovery from surgery [20, 21]. CRC survivors face distinct challenges, in some instances amplified by the presence of a stoma, primarily due to the specific impact of the disease on bowel function and the digestive system [22, 23]. Issues like constipation, diarrhoea, flatulence, incontinence, food intolerance, nausea, and loss of appetite present unique physical, psychological, and social challenges in following dietary and physical activity guidelines [20–24]. Access to effective post-treatment guidelines could play a role in reducing these sequalae and the potential influence on cancer control outcomes such as CRC recurrence and CRC-specific mortality [8, 15–19].

Greater internet access and usage over recent years has increased the number of people seeking post-treatment guidance online [25–28]. National cancer organisation (NCO) websites are frequently used as a source of information suggesting that survivors of CRC perceive these organisations as credible [26, 29, 30]. Previous studies report diet and physical activity advice for cancer primary prevention is well-addressed on NCO websites, yet the extent and suitability of guidance for CRC survivorship is limited [30, 31].

Previous studies have explored the opinions of survivors of CRC about nutrition advice received from healthcare professionals; investigated the availability, nature, and content of advice online for all survivors of cancer; and assessed the quality and useability of websites [26, 32, 33]. Whilst examining survivors’ perceptions of, and engagement with, recommendations on NCOs is critical, what is less clear is the extent to which such recommendations are grounded in evidence. Cancer survivors have reported using informal sources for dietary and physical activity advice, including social networks, the internet, magazines, and newspapers [25, 26, 31]. However, information from these sources can be inadequate, inconsistent, and on occasion inaccurate, suggesting there is a need for accurate, reliable, and evidence-based diet and physical activity information for cancer survivors [23, 31, 34].

To date, we are not aware of any work that has specifically synthesized post-treatment recommendations for survivors of CRC, an area where there is a recognised gap between information needs and provision. To address this, we conducted a critical synthesis of web-based guidance for CRC survivorship obtained from NCO websites to identify diet and physical activity recommendations currently accessible to survivors of CRC. We compared CRC survivorship guidance to cancer prevention recommendations, as survivors of CRC are advised to adhere to these more general guidelines [7].

At present, the term ‘cancer survivor’ or ‘cancer survivorship’ lacks a universal definition [35]. For this study, the definition proposed by Little et al. [36] was adopted:

Those people who have had a cancer, and who are living, at any period after treatment, apparently free of recurrent or persistent cancer [36]*.*

## Methods

A critical synthesis was conducted to examine NCO web-based diet and physical activity guidance for cancer prevention and survivorship. The aim was to identify recommendations currently advocated to survivors of CRC.

### NCO selection

The International Agency for Research on Cancer (IARC) GLOBOCAN database was used to identify countries with CRC incidence rates higher than the world average of 19.5 average standardised rate [37]. The review included English language NCO websites that ranked within the top 3 results on Google with the highest frequency for the identified search terms. These results are reported to account for the majority of users first clicks. Google was chosen, as this is the dominant search engine for the countries included in this study [38]. NCOs have been identified in previous studies as a common information source used by survivors of CRC [25, 26, 29, 30]. Search terms included “cancer survivorship”, “colorectal cancer”, “bowel cancer”, “diet”, “nutrition”, “exercise”, “physical activity”, “cancer organisation”, “cancer charity”, and “advice”. Each term was also combined with the name of the included countries. Major NCOs with a dominant online presence also appeared in the top 3 results outside of their country of origin. We excluded duplicate results and included the subsequent search results to get country-specific results. This search methodology aimed to ensure the representation of smaller national NCOs which did not appear in the top 3 results initially due to replication of international NCOs in the search results.

### Search strategy

Each NCO website was initially reviewed to become familiarised with the structure, content, and navigation. A systematic search was conducted on each NCO website for keywords related to diet and physical activity. Keyword selection for dietary factors was guided by WCRF/AICR recommendations for cancer prevention and survivorship [7] (Online Resource 1). Search terms included “diet”, “nutrition”, “wholegrain”, “vegetable”, “fruit”, “bean”, “legume”, “pulse”, “fish”, “dairy” “meat”, “red meat”, “processed meat”, “processed”, “preserved”, “fast food”, “fat”, “salt”, “alcohol”, “sugar”, “sweetened”, “supplement”, “vitamin”, “mineral”, “calcium”, “fibre”, “fluid”, “protein”, and “soy”. Keyword selection for components of physical activity included “aerobic activity”, “resistance training”, and “flexibility and balance”, as defined by the American College of Sports Medicine [39]. Additional search terms included “weight”, “activity”, “exercise”, “movement”, “training”, “aerobic”, “aerobic”, “resistance”, “flexibility”, “balance”, “stretching”, and “strength”.

Various search strategies were implemented to ensure comprehensive data collection [28]. These strategies included searching website menus and page links, utilising search bars within each website and using Google to search the name of each NCO in combination with the defined keywords. Searches were initially completed between the 10th and 24th of April 2022 and updated in June 2023.

### Data extraction

Data were collated and analysed using a Microsoft Access database. Data extracted included the following: (1) organisation name, (2) the dietary factor or physical activity related to the recommendation, and (3) aspects of the recommendation such as frequency, quantity, and duration. The extracted data also included information about the purpose of the recommendation, specifying whether it aimed to address (1) all cancer types or CRC specifically, (2) cancer prevention or survivorship, and (3) improved cancer-control outcomes or improved general health. For prevention, the cancer-control outcome was defined as reduced CRC risk. For survivorship, the cancer-control outcome was defined as reduced risk of CRC recurrence or CRC-specific mortality. The research team discussed ambiguous or inconsistent content to gain consensus on classification. Metadata were collected to allow the identification of source information.

Webpages were revisited if new categories were identified iteratively. Diet and physical activity content was included if relevant to cancer prevention or survivorship and integrated into the core website content, including online PDF booklets. The focus of this study was on the dietary and physical activity factors recommended to survivors of CRC to improve cancer-control outcomes, so content guiding the implementation of these recommendations was excluded. For example, practical, psychological, and financial topics were excluded, such as recipe suggestions, the impact of dietary changes on well-being, and food budget. Additionally, content accessed through external links, blog posts or news articles (due to their transient nature), and advice related specifically to treatment side effects were excluded.

### Data synthesis

We conducted an appraisal to assess the frequency of diet and physical activity guidance on each NCO website. We also examined the consistency of individual recommendations across different NCO websites. Following this appraisal, we conducted a synthesis of available recommendations for CRC survivorship to provide a comprehensive overview of public-facing diet and physical activity guidance for survivors of CRC.

## Results

A total of six English-speaking countries were included in the study: Ireland, UK, USA, Australia, New Zealand, and Canada. From these six countries, 13 NCOs met the initial selection criteria (Table 1). Diet and physical activity recommendations on the American Institute of Cancer Research (AICR) website were recorded but excluded from the results as we opted to use the joint WCRF/AICR Continuous Update Project Expert Report 2018 to inform the search terms for this synthesis [7]. This report is considered an extensive and authoritative review of the research on diet, physical activity, and cancer [7]. Furthermore, the report is referenced by several NCO websites as a source of information and is emerging as canonical. Twelve NCO websites were included in the final analysis (Table 1).

**Table 1:** National Cancer Organisations. English language NCO websites consistently in the top 3 results for search terms related to diet, nutrition, physical activity, and cancer on the Google search engine. *ASR*, age standardised incidence rate per 100,000. Source: GLOBOCAN 2020 [40]

Country	Cancer incidence (ASR)	National cancer organisation (NCO)	Website
Ireland	34.9	Irish Cancer Society	cancer.ie
UK	34.1	Cancer Research UK (CRUK)	cancerresearchuk.org
		Macmillan Cancer Support	macmillan.org.uk
		Bowel Cancer UK	bowelcanceruk.org.uk
New Zealand	33.8	Cancer Society New Zealand	cancer.org.nz
		Bowel Cancer New Zealand	bowelcancernz.org.nz
Australia	33.1	Cancer Council Australia	cancer.org.au
		Bowel Cancer Australia	bowelcanceraustralia.org
Canada	31.2	Canadian Cancer Society	cancer.ca
		Colorectal Cancer Canada	colorectalcancercanada.com
USA	25.6	American Cancer Society	cancer.org
		American Society of Clinical Oncology	cancer.net

NCO websites providing advice and guidance for all-cancer types were identified in each country. Each website provided CRC-specific information pages and were included in this study. In addition, in the UK, Australia, Canada, and New Zealand, NCO websites dedicated to providing CRC-advice and guidance were identified and included in this study.

A common structure was observed across these websites including sections dedicated to general cancer information, causes, risk factors, prevention, treatment, and survivorship. Each general NCO website had dedicated sections for specific cancer types, including CRC.

A total of 827 pages of content were reviewed. We identified more than 600 references to diet and physical activity and classified 27 distinct areas of diet and physical activity advice (Online Resource 2, Online Resource 3).

**Fig. 1:**
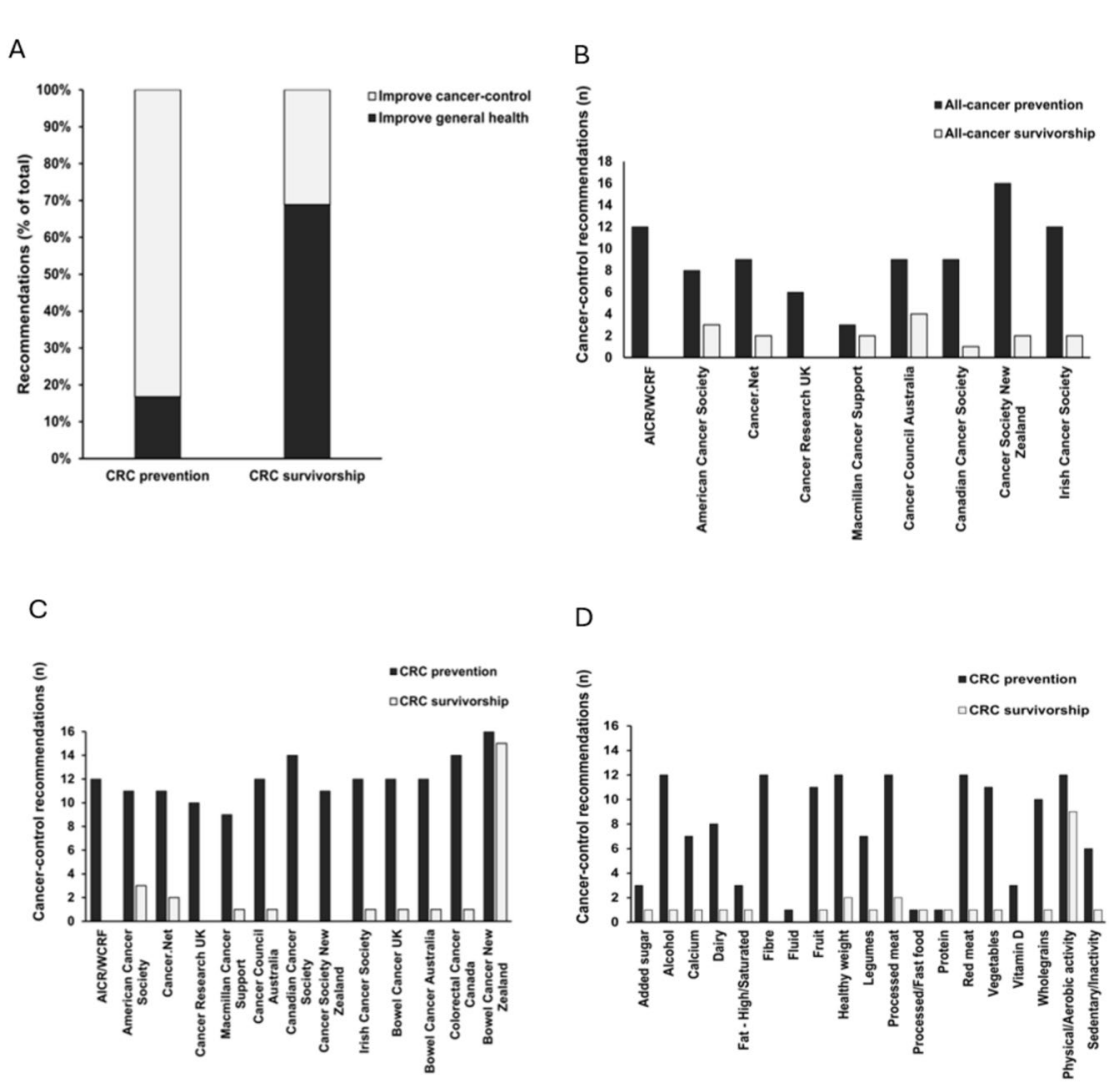
Frequency by which diet and physical activity recommendations are made on NCO websites. **A** Diet and physical activity recommendations by intended outcome (general health or cancer-control) and cancer phase (prevention or survivorship) for CRC across all NCO websites. **B** Frequency of distinct diet and physical activity recommendations for cancer-control by cancer phase and NCO for all-cancer. **C** Frequency of distinct diet and physical activity recommendations for cancer-control by cancer phase and NCO for CRC. **D** Frequency of distinct diet and physical activity recommendations for cancer-control by cancer phase and diet/physical activity factor for CRC

The objective of diet and physical activity recommendations varied depending on cancer phase and cancer type. For CRC prevention, cancer-control recommendations to reduce CRC risk (83%) were more frequent than recommendations to improve general health (17%). In contrast, for CRC survivorship recommendations to improve general health (69%) were more frequent than recommendations to improve cancer-control outcomes (31%) (Fig. 1A).

Diet and physical activity recommendations to improve cancer-control outcomes were primarily aimed at prevention (reducing risk) not survivorship (reducing recurrence or mortality) for both cancer in general (Fig. 1B) and CRC-specific cancer (Fig. 1C).

For CRC prevention, recommendations to improve cancer-control outcomes consistent across NCO websites included maintaining a healthy weight; increasing physical activity, intake of fibre, wholegrains, fruit, and vegetables; and reducing intake of alcohol and red and processed meat. For CRC survivorship, increased physical activity was the only recommendation to improve cancer-control outcomes consistent across NCO websites (Fig. 1D). Summarises the diet and physical activity factors recommended on at least half of the NCO websites to improve cancer-control outcomes for CRC prevention and survivorship (Table 2).

**Table 2:** Diet and physical activity recommendations to improve cancer-control outcomes for CRC prevention and survivorship available on at least half of the NCO websites

Diet/physical activity factor	Recommended action	CRC guidance for:	
		Prevention	Survival
Alcohol	Avoid/limit alcohol to 1 unit (F) or 2 units (M) a day, include at least 2 alcohol free days	Reduce risk	
Calcium	Eat a diet rich in calcium	Reduce risk	
Dairy	Consume at least 2 portions of dairy a day	Reduce risk	
Fibre (inc. fruit, vegetables, wholegrains, and legumes)	Consume 25–30 g of fibre and at least 5 portions of fruit and vegetables a day. Include wholegrains and legumes	Reduce risk	
Healthy weight	Maintain a healthy weight	Reduce risk	
Processed meat	Reduce/exclude processed meat	Reduce risk	
Red meat	Limit red meat to less than 500 g a week	Reduce risk	
Physically active (inc. aerobic)	Include 150 min moderate/75 min vigorous physical activity a week	Reduce risk	Reduce risk
Sedentary/inactivity	Avoid sedentary behaviour and inactivity	Reduce risk	

In summary, diet and physical activity content on NCO websites was organised according to cancer type, disease phase, and intended health objective. The primary objective of CRC survivorship guidance was to improve general health not cancer-control outcomes (CRC recurrence or CRC-specific mortality). In contrast, guidance for CRC prevention did focus on cancer-control outcomes (reduced CRC risk). Critical synthesis of the data confirmed that dietary recommendations aimed at improving cancer-control outcomes for CRC survivors were limited and often not evidence based. In contrast, physical activity guidance was evidence-based and consistent across organisations.

## Discussion

This study identified and synthesised diet and physical activity content available on NCO websites to determine the frequency and nature of recommendations designed to improve cancer-control outcomes for CRC survivors. We compared these findings to recommendations for cancer prevention as survivors of CRC are often advised to adhere to these guidelines, which may be, in part, due to an evidence shortfall in CRC survivorship research [41]. Dietary guidance on NCO websites is reflective of this and largely focused on general cancer guidance and CRC prevention. Overall, recommendations were consistent with conclusions from the WCRF/AICR Third Expert report for CRC prevention [41].

However, prevention advice may not be optimal for the information and support needs of survivors, and the WCRF/AICR Third Expert report concluded there was insufficient evidence to establish specific guidelines for CRC survivorship [41]. Survivors of CRC face unique challenges to the general cancer population in following diet and physical activity guidelines due the impact of the disease on digestion and bowel function [22–24]. Sequalae like constipation, diarrhoea, nausea, and loss of appetite can impact tolerance or digestion of foods recommended for prevention, along with uptake of physical activity, potentially influencing cancer control outcomes [8, 15–19]. To date, randomised control trials using diet and nutrition-based interventions derived from primary prevention evidence, have met with little success in the prevention of CRC recurrence and CRC-specific mortality [10], suggesting that a more tailored approach is required for survivors of CRC.

Content on one NCO website advises limiting red meat and avoiding processed meat to reduce the risk of recurrence, reflecting prevention advice for reduced CRC risk. However, following CRC prevention guidelines to reduce red meat, without adequate consideration of the nutritional value of alternative protein sources, could contribute to a deficiency of nutrients essential to the recovery process. Ford et al. [15] advise caution in applying prevention recommendations to reduce red meat consumption for cancer survivors, and it has been suggested that, for optimal muscle health, 65% of protein intake for cancer survivors should come from animal sources [15].

Recommendations to maintain a healthy weight can present unique challenges for CRC survivors, and evidence for an association between post-diagnostic BMI and CRC mortality is inconsistent [8]. Schlesinger et al. [42] found that compared to those with a healthy weight, CRC survivors who were overweight had a lower risk of all-cause mortality. Lee et al. [43] reflected this finding in a meta-analysis, which also reported a lower risk of CRC-specific mortality amongst patients who were overweight post-diagnosis. However, being underweight or obese post cancer diagnosis were associated with increased all-cause mortality, though no association was found for CRC-specific mortality. van Zutphen et al. [44] reported that post-diagnostic weight loss of over 10% was also associated with increased CRC-specific mortality. Factors such as weight stability, impact of long-term disease, treatment effects, and other health variables need to be considered when interpreting these reports. These nuances highlight the benefit of personalised support for tailored weight management during CRC survivorship [45].

Dairy products and calcium supplements are commonly advised for CRC prevention on several NCO websites, aligning with current evidence [41, 46] Low-fat dairy is often specified yet WCRF/AICR do not distinguish between types of dairy in their review of the evidence [41]. High-fat dairy consumption is one element of the Western dietary pattern associated with an increased risk of CRC recurrence [11] suggesting clarification on dairy type and an association with CRC recurrence and mortality is important.

Limiting alcohol consumption is recommended on each NCO websites to reduce the risk of cancer; however, whilst ten of the 12 NCO websites recommend limiting alcohol consumption to improve general health during survivorship, specific recommendations for reducing the risk of recurrence or mortality were not identified. At present, studies show inconsistent results for an association between alcohol intake and cancer survival outcomes [11, 47]. Results vary between cancer sites and pre-diagnostic or post-diagnostic consumption. Lower CRC-specific mortality is reported for light drinkers compared with non-drinkers [11, 47], though these results may be influenced by factors such as abstinence from alcohol due to underlying health issues.

A novel finding from this review is that physical activity (75 min of vigorous activity, 150 min of moderate activity a week, or an equivalent combination of both) is the only consistent recommendation on all NCO websites for improving cancer-control outcomes for CRC survivors. This guidance is supported by the current evidence-base, which shows that increased physical activity post-diagnosis is associated with lower CRC-specific mortality [8, 16, 41, 48, 49]. Qiu et al. reported a 24% lower risk of CRC-specific mortality for each additional 10 MET hours per week of post-diagnosis physical activity [50], which is equivalent to approximately 150 min of brisk walking per week.

In contrast to the findings in this review, a previous study reported dietary information for survivors is well-addressed on NCO websites [29]. Whilst we would agree that there is easily accessible and comprehensive advice about diet and physical activity, within dedicated survivorship sections of many NCO websites, the guidance is frequently generic, derived from prevention guidance, and not evidence-based guidance for CRC survivors. Misinformation in the media and online is reported as a barrier to adherence to diet and physical activity guidelines [25, 32, 51]. NCOs are seen as a reliable source of information [25, 26, 52]. This suggests a need for accurate, evidence-based diet and physical activity information specifically for CRC survivors on NCO websites.

Our study specifically explores recommendations to improve cancer-control outcomes for survivors of CRC, an area where access to diet and physical activity advice has been shown to be limited [29]. Clarity regarding the intended audience for guidance and the objective of recommendations is important, not least because the guidance for prevention may not meet the needs of CRC survivors [8, 15, 16].

Our findings support a recent paper by Keaver et al. [31] which concludes that overall nutritional content for cancer survivors is limited across a range of websites that offer diet and physical activity guidance. This report emphasises the importance of tailoring dietary advice to different cancer types, and our research provides a comprehensive evaluation of the availability of guidance specifically for CRC survivorship.

### Study limitations

Limitations of this study include the exclusion of blogs and news articles due to the high volume and topical content, inclusion of opinion pieces, potential website updates since completion of the searches, and inherent subjectivity in the interpretation of website content. The systematic search approach employed for this study could also be a limitation, as searches conducted by cancer survivors may be more organic and produce different results. However, our approach also lends strengths to the study as we repeatedly reviewed core content across entire websites, used multiple search techniques to mitigate individuality in search methods, and collaborated across the research team to reduce subjectivity in interpretation. This is the first study to evaluate web-based diet and physical activity recommendations specifically for survivors of CRC post-treatment.

## Conclusion

- Diet and physical activity guidance available on NCO websites is easily accessible and offers recommendations for improving general health during CRC survivorship; recommendations for physical activity are consistent and in-line with the evidence-base.
- However, dietary guidance aimed at improving recurrence and mortality in CRC survivors is inadequate, reflecting the currently limited evidence base, upon which to develop tailored guidelines for this population.
- Diet and physical activity guidance needs to be tailored to the type of cancer diagnosed, and the disease phase being addressed. Recommendations for CRC survivors need to be clear, consistent, and relevant to their specific requirements.
- The evidence shortfall in CRC survivorship must be addressed to enable delivery of these recommendations.

## Supplementary Information

Below is the link to the electronic supplementary material.

Supplementary file1 (DOCX 334 KB)

## Data Availability

Data, in the form of an excel file, is available from the corresponding author upon request.

## References

[1] National Institute for Health and Care Excellence (2020) Colorectal cancer. NICE guideline 151. In: National Institute for Health and Care Excellence. https://www.nice.org.uk/guidance/ng151. Accessed 28 Jun 2022

[2] van Veen MR, Winkels RM, Janssen SHM et al (2018) Nutritional information provision to cancer patients and their relatives can promote dietary behavior changes independent of nutritional information needs. Nutr Cancer 70:483–489. 10.1080/01635581.2018.1446092 29537887 10.1080/01635581.2018.1446092

[3] van Zutphen M, Boshuizen HC, Kok DE et al (2019) Colorectal cancer survivors only marginally change their overall lifestyle in the first 2 years following diagnosis. J Cancer Surviv 13:956–967. 10.1007/s11764-019-00812-7 31646463 10.1007/s11764-019-00812-7 PMC6881417

[4] Trujillo EB, Claghorn K, Dixon SW et al (2019) Inadequate nutrition coverage in outpatient cancer centers: results of a national survey. J Oncol 2019:7462940. 10.1155/2019/7462940 31885583 10.1155/2019/7462940 PMC6893237

[5] Maunsell R, Sodergren S, Hopkinson J et al (2021) Nutritional care in colorectal cancer-what is the state of play? Colorectal Dis. 10.1111/codi.15933 34605160 10.1111/codi.15933

[6] Orange ST, Gilbert SE, Brown MC, Saxton JM (2021) Recall, perceptions and determinants of receiving physical activity advice amongst cancer survivors: a mixed-methods survey. Support Care Cancer 29:6369–6378. 10.1007/s00520-021-06221-w 33885962 10.1007/s00520-021-06221-w PMC8464579

[7] World Cancer Research Fund/American Institute Cancer Research (2018) Food, nutrition, physical activity, and the prevention of cancer: a global perspective. Continuous Update Project Expert Report

[8] Rock CL, Thomson CA, Sullivan KR et al (2022) American Cancer Society nutrition and physical activity guideline for cancer survivors. CA Cancer J Clin. 10.3322/caac.21719 35294043 10.3322/caac.21719

[9] Moazzen S, van der Sloot KWJ, de Bock GH, Alizadeh BZ (2021) Systematic review and meta-analysis of diet quality and colorectal cancer risk: is the evidence of sufficient quality to develop recommendations? Crit Rev Food Sci Nutr 61:2773–2782. 10.1080/10408398.2020.1786353 32613845 10.1080/10408398.2020.1786353

[10] Van Blarigan EL, Meyerhardt JA (2015) Role of physical activity and diet after colorectal cancer diagnosis. J Clin Oncol 33:1825–1834. 10.1200/JCO.2014.59.7799 25918293 10.1200/JCO.2014.59.7799 PMC4438267

[11] Schwedhelm C, Boeing H, Hoffmann G et al (2016) Effect of diet on mortality and cancer recurrence among cancer survivors: a systematic review and meta-analysis of cohort studies. Nutr Rev 74:737–748. 10.1093/nutrit/nuw045 27864535 10.1093/nutrit/nuw045 PMC5181206

[12] Muscaritoli M, Arends J, Bachmann P et al (2021) ESPEN practical guideline: clinical nutrition in cancer. Clin Nutr 40:2898–2913. 10.1016/j.clnu.2021.02.005 33946039 10.1016/j.clnu.2021.02.005

[13] Demark-Wahnefried W, Aziz NM, Rowland JH, Pinto BM (2005) Riding the crest of the teachable moment: promoting long-term health after the diagnosis of cancer. J Clin Oncol 23:5814–5830. 10.1200/JCO.2005.01.230 16043830 10.1200/JCO.2005.01.230 PMC1550285

[14] Sullivan ES, Rice N, Kingston E et al (2021) A national survey of oncology survivors examining nutrition attitudes, problems and behaviours, and access to dietetic care throughout the cancer journey. Clin Nutr ESPEN 41:331–339. 10.1016/j.clnesp.2020.10.023 33487286 10.1016/j.clnesp.2020.10.023

[15] Ford KL, Arends J, Atherton PJ et al (2022) The importance of protein sources to support muscle anabolism in cancer: an expert group opinion. Clin Nutr 41:192–201. 10.1016/j.clnu.2021.11.032 34891022 10.1016/j.clnu.2021.11.032

[16] Song R, Petimar J, Wang M et al (2021) Adherence to the World Cancer Research Fund/American Institute for Cancer Research Cancer Prevention Recommendations and Colorectal Cancer Survival. Cancer Epidemiol Biomark Prev 30:1816–1825. 10.1158/1055-9965.epi-21-0120 10.1158/1055-9965.EPI-21-0120 PMC8492523 34272268

[17] Keaver L, Houlihan C, O’Callaghan N et al (2021) Evidence-based nutrition guidelines for cancer survivors in Europe: a call for action. Eur J Clin Nutr. 10.1038/s41430-021-01036-8 34716363 10.1038/s41430-021-01036-8

[18] Mols F, Beijers T, Lemmens V et al (2013) Chemotherapy-induced neuropathy and its association with quality of life among 2- to 11-year colorectal cancer survivors: results from the population-based PROFILES registry. J Clin Oncol 31:2699–2707. 10.1200/JCO.2013.49.1514 23775951 10.1200/JCO.2013.49.1514

[19] Thong MSY, Mols F, Wang XS et al (2013) Quantifying fatigue in (long-term) colorectal cancer survivors: a study from the population-based patient reported outcomes following initial treatment and long term evaluation of survivorship registry. Eur J Cancer 49:1957–1966. 10.1016/j.ejca.2013.01.012 23453750 10.1016/j.ejca.2013.01.012 PMC3676930

[20] Emery J, Butow P, Lai-Kwon J et al (2022) Management of common clinical problems experienced by survivors of cancer. The Lancet 399:1537–1550. 10.1016/S0140-6736(22)00242-2 10.1016/S0140-6736(22)00242-2 35430021

[21] Denlinger C, Barsevick AM, Vega CP (2009) The challenges of colorectal cancer survivorship. J Natl Compr Cancer Netw 7:883–894 10.6004/jnccn.2009.0058 PMC3110673 19755048

[22] Drury A, Payne S, Brady AM (2017) Cancer survivorship: advancing the concept in the context of colorectal cancer. Eur J Oncol Nurs 29:135–147. 10.1016/j.ejon.2017.06.006 28720260 10.1016/j.ejon.2017.06.006

[23] Beaver K, Latif S, Williamson S et al (2010) An exploratory study of the follow-up care needs of patients treated for colorectal cancer. J Clin Nurs 19:3291–3300. 10.1111/j.1365-2702.2010.03407.x 20964750 10.1111/j.1365-2702.2010.03407.x

[24] Anderson AS, Steele R, Coyle J (2013) Lifestyle issues for colorectal cancer survivors—perceived needs, beliefs and opportunities. Support Care Cancer 21:35–42. 10.1007/s00520-012-1487-7 22773297 10.1007/s00520-012-1487-7

[25] Beeken RJ, Williams K, Wardle J, Croker H (2016) “What about diet?” A qualitative study of cancer survivors’ views on diet and cancer and their sources of information. Eur J Cancer Care (Engl) 25:774–783. 10.1111/ecc.12529 27349812 10.1111/ecc.12529 PMC4995727

[26] Matsell SL, Sánchez-García MA, Halliday V et al (2020) Investigating the nutritional advice and support given to colorectal cancer survivors in the UK: is it fit for purpose and does it address their needs? J Hum Nutr Diet 33:822–832. 10.1111/jhn.12815 32951269 10.1111/jhn.12815

[27] Ebel MD, Stellamanns J, Keinki C et al (2017) Cancer patients and the internet: a survey among german cancer patients. J Cancer Educ 32:503–508. 10.1007/s13187-015-0945-6 26553327 10.1007/s13187-015-0945-6

[28] Wang X, Shi J, Kong H (2021) Online health information seeking: a review and meta-analysis. Health Commun 36:1163–1175. 10.1080/10410236.2020.1748829 32290679 10.1080/10410236.2020.1748829

[29] Barrett M, Uí Dhuibhir P, Njoroge C et al (2020) Diet and nutrition information on nine national cancer organisation websites: a critical review. Eur J Cancer Care (Engl) 29:1–22. 10.1111/ecc.13280 10.1111/ecc.13280 32639069

[30] Llaha F, Ribalta A, Arribas L et al (2022) A review of web-based nutrition information in Spanish for cancer patients and survivors. Nutrients 14(7):1441. 10.3390/nu14071441 35406054 10.3390/nu14071441 PMC9003392

[31] Keaver L, Huggins MD, Chonaill DN, O’Callaghan N (2023) Online nutrition information for cancer survivors. J Hum Nutr Diet 36:415–433. 10.1111/jhn.13095 36177612 10.1111/jhn.13095

[32] Hardcastle SJ, Maxwell-Smith C, Hagger MS et al (2018) Exploration of information and support needs in relation to health concerns, diet and physical activity in colorectal cancer survivors. Eur J Cancer Care (Engl) 27(1):e12679. 10.1111/ecc.12679 10.1111/ecc.12679 28337818

[33] National Institute for Health Research (2015) NIHR Cancer and Nutrition Infrastructure Collaboration, Report of Phase One. https://cancerandnutrition.nihr.ac.uk/wpcontent/uploads/2016/06/Cancer-Nutrition-Full-Report-FINAL_03-06-16.pdf. Accessed 16 Aug 2022

[34] Goldacre B (2009) Media misinformation and health behaviours. Lancet Oncol 10:848. 10.1016/S1470-2045(09)70252-9 19717089 10.1016/S1470-2045(09)70252-9

[35] Marzorati C, Riva S, Pravettoni G (2017) Who is a cancer survivor? A systematic review of published definitions. J Cancer Educ 32:228–237. 10.1007/s13187-016-0997-2 26854084 10.1007/s13187-016-0997-2

[36] Little M, Sayers EJ, Paul K, Jordens CF (2000) On surviving cancer. J R Soc Med 93:501 11064684 10.1177/014107680009301001 PMC1298120

[37] Ferlay J, M E, Lam F et al (2020) Estimated number of new cases in 2020, all cancers, both sexes, all ages. https://gco.iarc.fr/today/online-analysis-table?v=2020&mode=population&mode_population=countries&population=900&populations=900&key=asr&sex=0&cancer=39&type=0&statistic=5&prevalence=0&population_group=0&ages_group%5B%5D=0&ages_group%5B%5D=17&group_cancer=. Accessed 21 Apr 2022

[38] Gs.statcounter.com (2022) Search Engine Market Share. Aug 2021 - Sept 2022. In: Statcounter GlobalStats. https://gs.statcounter.com/search-engine-market-share/all. Accessed 20 Oct 2022

[39] Garber CE, Blissmer B, Deschenes MR et al (2011) Quantity and quality of exercise for developing and maintaining cardiorespiratory, musculoskeletal, and neuromotor fitness in apparently healthy adults: guidance for prescribing exercise. Med Sci Sports Exerc 43:1334–1359. 10.1249/MSS.0b013e318213fefb 21694556 10.1249/MSS.0b013e318213fefb

[40] Ferlay J, Ervik M, Lam F et al (2020) Global cancer observatory: cancer today. In: International Agency for Research on Cancer. https://gco.iarc.fr/today/about. Accessed 26 Jun 2022

[41] World Cancer Research Fund/American Institute for Cancer Research (2018) Continuous update project expert report 2018. Diet, nutrition, physical activity and colorectal cancer

[42] Schlesinger S, Siegert S, Koch M et al (2014) Postdiagnosis body mass index and risk of mortality in colorectal cancer survivors: a prospective study and meta-analysis. Cancer Causes Control 25:1407–1418. 10.1007/s10552-014-0435-x 25037235 10.1007/s10552-014-0435-x

[43] Lee J, Jeon JY, Meyerhardt JA (2015) Diet and lifestyle in survivors of colorectal cancer. Hematol Oncol Clin North Am 29:1–27. 10.1016/j.hoc.2014.09.005. (PT - Journal Article, Review) 25475570 10.1016/j.hoc.2014.09.005 PMC4258898

[44] van Zutphen M, Kampman E, Giovannucci EL, van Duijnhoven FJB (2017) Lifestyle after colorectal cancer diagnosis in relation to survival and recurrence: a review of the literature. Curr Colorectal Cancer Rep 13:370–401. 10.1007/s11888-017-0386-1 29104517 10.1007/s11888-017-0386-1 PMC5658451

[45] Barlow KH, van der Pols JC, Ekberg S, Johnston EA (2022) Cancer survivors’ perspectives of dietary information provision after cancer treatment: a scoping review of the Australian context. Health Promot J Austr 33:232–244. 10.1002/hpja.496 33890348 10.1002/hpja.496

[46] Vieira AR, Abar L, Chan DSM et al (2017) Foods and beverages and colorectal cancer risk: a systematic review and meta-analysis of cohort studies, an update of the evidence of the WCRF-AICR Continuous Update Project. Ann Oncol 28:1788–1802. 10.1093/annonc/mdx171 28407090 10.1093/annonc/mdx171

[47] Kim Y, Je Y, Giovannucci EL (2019) Association between alcohol consumption and survival in colorectal cancer: a meta-analysis. Cancer Epidemiol Biomark Prev 28:1891–1901. 10.1158/1055-9965.EPI-19-0156 10.1158/1055-9965.EPI-19-0156 31399475

[48] van Blarigan EL, Fuchs CS, Niedzwiecki D et al (2018) Association of survival with adherence to the American Cancer Society nutrition and physical activity guidelines for cancer survivors after colon cancer diagnosis: the CALGB 89803/alliance trial. JAMA Oncol 4:783–790. 10.1001/jamaoncol.2018.0126 29710284 10.1001/jamaoncol.2018.0126 PMC6145685

[49] Patel AV, Friedenreich CM, Moore SC et al (2019) American College of Sports Medicine roundtable report on physical activity, sedentary behavior, and cancer prevention and control. Med Sci Sports Exerc 51:2391–2402. 10.1249/MSS.0000000000002117 31626056 10.1249/MSS.0000000000002117 PMC6814265

[50] Qiu S, Jiang C, Zhou L (2020) Physical activity and mortality in patients with colorectal cancer: a meta-analysis of prospective cohort studies. Eur J Cancer Prev 29(1):15–26 30964753 10.1097/CEJ.0000000000000511

[51] Maxwell-Smith C, Zeps N, Hagger MS et al (2017) Barriers to physical activity participation in colorectal cancer survivors at high risk of cardiovascular disease. Psychooncology 26:808–814. 10.1002/pon.4234 27478009 10.1002/pon.4234

[52] Keaver L, O’Callaghan N, Douglas P (2022) Nutrition support and intervention preferences of cancer survivors. J Hum Nutr Diet. 10.1111/jhn.13058 35778782 10.1111/jhn.13058

