# Supplementary material for: Diet and physical activity advice for colorectal cancer survivors: critical synthesis of public-facing guidance
Source: Support Care Cancer. 2024 Aug 22;32(9):609. doi: 10.1007/s00520-024-08797-5 (PMC11341579; doi:10.1007/s00520-024-08797-5)
Supplement: Supplementary file 1 — Supplementary file1 (DOCX 334 KB) [file 520_2024_8797_MOESM1_ESM.docx]

# Diet and physical activity advice for colorectal cancer survivors: Critical synthesis of public-facing guidance

**Online Resources**

**Online Resource 1** A summary of WCRF/AICR diet, weight and physical activity recommendations [26]. for all-cancer prevention including the strength of evidence of each recommendation for CRC incidence published in the Diet, Nutrition and Physical Activity: a Global Perspective report[7]. The report considers ten lifestyle factors in relation to cancer risk developed from continuous analysis of the latest cancer prevention and survivorship research. It is accompanied by the Diet, Nutrition, Physical Activity and Colorectal Cancer report.

| **Lifestyle factor** | **Recommendations** | **Strength of evidence** |
| --- | --- | --- |
| *All-cancer prevention* | | |
| Wholegrains, vegetables, fruit | Eat wholegrains, vegetables, fruit and beans | Strong/probable (wholegrains) |
| Meat, fish and dairy products | Limit processed and red meat | Strong/probable |
| Preserved/processed food | Limit fast food | n/a |
| Non-alcoholic drinks | Limit sugar sweetened drinks | n/a |
| Alcoholic drinks | Limit alcohol consumption | Strong/convincing |
| Other dietary exposures | Don’t use supplements for cancer prevention | n/a |
| Physical activity | Be physically active | Strong/convincing |
| Body fatness | Be a healthy weight | Strong/convincing |
| Height and birth weight | n/a | Strong/convincing |
| Lactation | Breastfeed your baby, if you can | n/a |
| Cancer survivors | After diagnosis follow these guidelines, if you can. | n/a |
| *Additional factors for CRC prevention* | | |
| Calcium | Consume | Strong/probable |
| Fibre | Consume | Strong/probable |
| Dairy | Consume | Strong/probable |

**Online Resource 2** A summary of outcome-focused diet and physical activity recommendations available on NCO websites for both all-cancer and CRC prevention and survivorship. Shaded boxes indicate a dietary or physical activity recommendation identified on the corresponding NCO website for the indicated cancer type and phase (all-cancer prevention, all-cancer survivorship, CRC prevention, CRC survivorship). For each dietary or physical activity factor the recommended behaviour was dichotomised into reduce or include

**Online Resource 3** A summary of diet and physical activity recommendations aimed at improving general health available on NCO websites for both all-cancer and CRC prevention and survivorship. Shaded boxes indicate a dietary or physical activity recommendation identified on the corresponding NCO website for the indicated cancer type and phase (all-cancer prevention, all-cancer survivorship, CRC prevention, CRC survivorship). For each dietary or physical activity factor the recommended behaviour was dichotomised into reduce or include
